# Parental depression and child temperament: Assessing child to parent effects in a longitudinal population study

**DOI:** 10.1016/j.infbeh.2009.11.004

**Published:** 2010-02

**Authors:** Lucy Hanington, Paul Ramchandani, Alan Stein

**Affiliations:** University of Oxford Department of Psychiatry, Warneford Hospital, Headington, Oxford OX3 7JX, UK

**Keywords:** Temperament, Maternal depression, Paternal depression, ALSPAC

## Abstract

Current research supports a link between maternal depression and difficult child temperament. The direction of effect is often assumed to be from parent to child, but few studies have addressed child to parent effects. In a large cohort study, the Avon Longitudinal Study of Parents and Children (ALSPAC) (*N* = 14663), we aimed to further existing knowledge by investigating the relationship between maternal and paternal depressive symptoms and child temperament, and determining the direction of any effects found. Data was collected at 2 time-points (when the children were 6 and 24 months old), using the Edinburgh Postnatal Depression Scale and the Mood and Intensity subscales of the Carey Temperament Scales. Significant parent to child effects were seen, with maternal and paternal depressive symptoms at Time 1 leading to more difficult temperament at Time 2. Father to child effects were significant only in male children. Little evidence was found for child to parent effects.

## 1 Introduction

The adverse effects of maternal depression (particularly postnatal depression) on offspring development have been widely documented in both the human and the animal literature. Children of depressed mothers are more likely to have cognitive, behavioural and attachment difficulties (Beardslee, Versage, & Gladstone, 1998; Murray, 1992; Murray, Fiori-Cowley, & Hooper, 1996a; Murray, Stanley, Hooper, King, & Fiori-Cowley, 1996b; Martins & Gaffan, 2000), and they are also at increased risk of impaired physical development (O’Brien, Heycock, Hanna, Jones, & Cox, 2004).

Although estimates of the incidence of paternal depression during the first year postpartum range from 1.2 to 25.5% (with higher rates being found if the mother is depressed) (Goodman, 2004), the effects of paternal depression on child development have received less attention. However, existing research suggests that child outcomes are affected. One large prospective population study demonstrated a significant association between paternal postnatal depression and adverse behavioural and emotional outcomes in offspring at 3.5 years, as well as a higher risk of conduct-related problems in boys (Ramchandani, Stein, Evans, & O’Connor, 2005). In addition, adolescent children have been shown to have increased rates of psychopathology if their father is depressed (Kane & Garber, 2004).

Current research also supports a link between parental psychiatric disorder and child temperament. Temperament can be defined as “constitutionally based differences in reactivity and self-regulation, in the domains of affect, activity and attention” (Rothbart & Bates, 2006). The definition of temperament has attracted controversy over the years. In their classic work, Thomas, Chess, Birch, Hertzig, and Korn (1963) described nine key dimensions of temperament, namely Activity, Adaptability, Approach, Distractibility, Intensity, Mood, Persistence, Rhythmicity and Threshold. They went on to describe temperament as being a genetically determined characteristic which exhibits stability over time. Subsequent work has led to re-categorisation of these dimensions into broader constructs, including Surgency/Extraversion (broadly equivalent to Positive Emotionality (PE)), Negative Emotionality (NE) and Effortful Control (Rothbart & Bates, 2006). Although temperament is considered to be constitutionally based and does exhibit a degree of stability over time, it is now recognised that it can be affected by experience, genetic heritage and maturation. Parental functioning and the family environment therefore have the potential to influence child temperament. Temperament, in turn, has the potential to mould individual adaptation to the environment (Clark & Watson, 1999; Rothbart & Bates, 2006), and thus has been implicated as a risk factor for psychopathology. The tripartite model developed by Clark and Watson (1991) is the most prominent theoretical model linking temperament and mood disorders. The model proposes that depression is characterised by low PE and high NE, whereas physiological overarousal and high NE are seen in anxiety, and it is supported by a number of self-report studies in both the adult (Brown, Chorpita, & Barlow, 1998; Watson, Clark, & Carey, 1988) and child (Chorpita & Daleiden, 2002; Lonigan, Carey, & Finch, 1994) literature.

Research has shown that offspring of depressed parents generally exhibit more difficult temperamental characteristics (Beck, 1996; Bruder-Costello et al., 2007; Cutrona & Troutman, 1986), and longitudinal studies suggest that difficult child temperament is linked with depression in the child in later life. For example, behavioural apathy in children has been linked with depression in both childhood and adulthood (van Os, Jones, Lewis, Wadsworth, & Murray, 1997), and children who demonstrate low extraversion are more likely to exhibit depressive symptoms at 18 years of age (Block, Gjerde, & Block, 1991). Behavioural ratings showing high levels of social reticence, inhibition and fear of novelty at age 3 have also been linked with increased risk of later mood disorder (Caspi, Moffitt, Newman, & Silva, 1996). The mechanisms underlying these links are not known. It is possible that certain temperamental characteristics may represent the means by which depression passes from generation to generation (Costello et al., 2002). Alternatively, such characteristics may alter the way in which individuals react to the environment or process information, with depressogenic consequences (Davidson et al., 2002). It is known that depressed parents show deficits in interactions with their children, and these deficits may have detrimental effects on child development. Work to date has tended to focus on the link between maternal depression and child temperament, and although one community-based study has found that low positive emotionality in children is associated with maternal, but not with paternal, mood disorder (Durbin, Klein, Hayden, Buckley, & Moerk, 2005), the population of fathers with a history of mood disorder was small (*n* = 12). The current study looks to expand on existing knowledge by looking at both maternal and paternal depressive symptoms and their links with child temperament (concentrating in particular on traits related to positive emotionality) using a large, population-based sample.

Although research on the link between child temperament and parental depression dates back over 20 years, few studies have sought to determine whether parental depression leads to difficult child temperament or whether the reverse is true (Bell, 1968). The question is of clinical relevance, as interventional strategies have tended to focus on ameliorating the impact of parental disorder on child development (for example by implementing programmes to identify and treat postnatal depression), whereas a different approach would be required to tackle the consequences of child to parent effects. There is some evidence that both mother to child and child to mother effects may be important; it has been shown that mothers of infants with difficult temperaments are at greater risk of depression (Murray et al., 1996a,b; Whiffen & Gotlib, 1989), and reciprocal relationships have been found between maternal depression and child rhythmicity and attention span/persistence (Sugawara, Kitamura, Toda, & Shima, 1999). We are not aware of any studies that have examined the direction of effect in fathers. We therefore aimed to test the hypothesis that both parent to child and child to parent effects exist, with depressive symptoms in a mother or father leading to difficult child temperament and vice versa.

## 2 Methods

### 2.1 Participants

The Avon Longitudinal Study of Parents and Children (ALSPAC) (Golding, Pembrey, & Jones, 2001) is a large longitudinal cohort study designed to collect a wide range of data on parents and their children from early pregnancy onwards. Pregnant women who were resident in Avon and had an expected delivery date between 1 April 1991 and 31 December 1992 were eligible to participate. The initial sample consisted of 14,541 pregnant women, with 14,676 foetuses. There were 14,062 live births and 13,988 children were alive at 1 year. Ethical approval for the study was obtained from the ALSPAC Law and Ethics Committee and the Local Research Ethics Committees.

Several studies have been carried out to assess the representativeness of the ALSPAC sample as compared to the populations of both the area of Avon and the UK. As has been the case in other cohorts, the ALSPAC sample has a slight deficit in ethnic minority and less affluent families (www.alspac.bristol.ac.uk). 51.7% of the children in the study were male and 48.3% were female. The majority of parents in the sample were white (97.4% of mothers and 96% of fathers). 1.6% of fathers were Black Caribbean, but no other single ethnic group contributed to more than 1% of the population of fathers or mothers. 95% of the children were classed as white. With regard to the mother's highest educational attainment, 64.7% of mothers had O levels (taken at age 16) or less, whereas 12.9% reported having a degree. In the UK, social class is occupationally defined. There are five categories ranging from I (professional) to V (unskilled). The majority of the mothers in the ALSPAC sample (50.6%) fell into social class III (skilled), with only 5.9% of mothers in social class I and 2.2% in social class V. A full breakdown of the sociodemographic characteristics of the sample is available from the authors.

### 2.2 Measures

Data on parental depression and child temperament were collected at two time-points: Time 1 (6–8 months after the birth of the child) and Time 2 (21–24 months after the birth of the child).

Mothers and fathers completed the Edinburgh postnatal depression scale (EPDS) at 8 months and 21 months after the birth of their baby. The EPDS is a ten-item self-report questionnaire which has been validated for use in mothers both during and after the postnatal period, and also in men (Cox, Holden, & Sagovsky, 1987; Cox, Chapman, Murray, & Jones, 1996; Matthey, Barnett, Kavanagh, & Howie, 2001). Although the EPDS is not a diagnostic tool, it has been shown that scores of greater than 12 have a high sensitivity (81.1%) and specificity (95.7%) in predicting major depressive disorder (Murray & Carothers, 1990). In this study the EPDS data for both mothers and fathers was split so that parents scoring more than 12 were categorised as ‘depressed’, whereas those scoring 12 or less were categorised as ‘not depressed’. Dichotomising the data in this way helped to ensure the clinical relevance of the results.

Child temperament was measured at 6 months and 24 months of age using the Carey Temperament Scales (CTSs) (Carey & McDevitt, 1978), which comprise a number of age-appropriate questions. These questions assess the nine temperamental characteristics first described by Thomas et al. (1963). Caregivers are presented with a statement describing a certain behaviour (for example “She lies quietly in the bath”) and asked to rate how often their child behaves in that way on a scale ranging from 1 (almost never) to 6 (almost always). The results are recoded so that higher scores indicate more difficult temperament. In the current study, mothers were responsible for completing the questionnaires, which were administered by post. To avoid the potential problems of repeated statistical testing, two of the nine dimensions of temperament were selected for analysis *a priori*. ‘Intensity’ and ‘Mood’ were chosen as they correlate most closely with the concept of positive emotionality, and previous research has shown an association between low positive emotionality in children and maternal mood disorder (Durbin et al., 2005). The ‘Mood’ subscale is designed to measure the general tone of affect (whether positive or negative overall), and the ‘Intensity’ subscale is designed to capture the level of energy with which an emotional response is made. The scales include items such as “She responds intensely (screams, yells) when frustrated” (Intensity), and “She frowns or complains when left to play alone” (Mood).

### 2.3 Statistical analysis

The analyses were undertaken in the following stages:

1. Linear regression analyses were carried out to assess the strength of the relationship between paternal depression at Time 1 and child temperament at Time 2. Separate analyses were carried out for the mood data and the intensity data, and these were repeated, controlling first for the effects of child temperament at Time 1 and then for paternal depression at Time 2. Similar analyses were then carried out using the maternal depression data.
2. Logistic regression analyses were carried out to assess the relationship between child temperament (looking at mood and intensity separately) at Time 1 and paternal depression at Time 2, and again these analyses were repeated, controlling first for the effects of child temperament at Time 2 and then for paternal depression at Time 1. These logistic regression analyses were then repeated for maternal depression.
3. The analyses were repeated for boys and girls separately to check for specificity of effects in relation to the sex of the child.
4. Finally, both maternal and paternal depression were entered into regression models together to assess the strengths of the independent effects of each.

All analyses were conducted using SPSS for Windows version 15.

## 3 Results

Overall, response rates were highest for the child-related and maternal measures, with data available on 10,317 (73.4%) children and 10,401 (74.0%) mothers at Time 2. The response rates were lower for paternal measures (6170 (43.9%) available at Time 2). Nonetheless the sample sizes remained large (see Table 1). Information on attrition from the ALSPAC study is available at www.alspac.bris.ac.uk.

### 3.1 Prevalence of maternal and paternal depression

In accordance with findings from previous epidemiological studies, rates of depression in the postnatal period were higher in mothers than fathers. Of the 11,333 mothers who completed the Edinburgh Postnatal Depression scale at Time 1, 998 (8.8%) were depressed. At Time 2, 1027 out of 10401 mothers were depressed (9.9%). By comparison, 3% of fathers (212 out of 7168) were depressed at Time 1 and 3.5% (216 out of 6170) of fathers were depressed at Time 2. Overall, the rates of maternal postnatal depression in this community sample were slightly lower than the rate (13%) calculated by O’Hara and Swain (1996) in their meta-analysis. The rates of paternal depression were comparable to those found in previous community studies (Goodman, 2004).

### 3.2 Paternal depression models

On average, children of fathers who were depressed at Time 1 were found to have significantly higher mood and intensity scores at Time 2 than children of fathers who were not depressed at Time 1. Paternal depression at Time 1 significantly predicted both child mood (Beta = 0.049; *p* < 0.001) and intensity (Beta = 0.038; *p* = 0.003) at Time 2. When the data were split according to gender, these father to child effects were found to be significant only for male children (see Table 2). In the case of male children, the effects remained significant even when mediation analyses controlling for (i) earlier mood/intensity and (ii) later paternal depression were carried out.

Child temperament (mood and intensity) scores at Time 1 did not predict paternal depression at Time 2, even when data were split according to gender (see Table 3). Therefore, no child to parent effects were demonstrated for child temperament and paternal depression.

### 3.3 Maternal depression models

Maternal depression at Time 1 predicted child mood at Time 2 (standardised coefficient Beta 0.113; *p* < 0.001) (see Table 4). This relationship remained significant when child mood at Time 1 was included in the regression analysis (standardised coefficient Beta 0.081; *p* < 0.001), and when maternal depression at Time 2 was included (standardised coefficient Beta 0.081; *p* < 0.001). When the above regression models were repeated, substituting child intensity at Time 2 for child mood at Time 2, similar results were found, with maternal depression at Time 1 significantly predicting higher child intensity scores (Beta = 0.088; *p* < 0.001). This association remained even when later maternal depression scores and earlier child intensity scores were controlled for.

Repeating the analyses using data split according to gender showed that maternal depression at Time 1 significantly predicted child temperament at Time 2 for both genders.

Unadjusted regression analyses revealed that both child mood (Beta = 0.020; *p* = 0.001) and intensity scores (Beta = 0.014; *p* = 0.026) at Time 1 significantly predicted later maternal depression (Time 2) (see Table 5). When the data were split according to gender, it was found that these effects were due mainly to the influence of female children on their mothers. However, when either earlier maternal depression scores, or later mood or intensity scores were included in the regression analyses the associations were substantially attenuated, suggesting that continuity of these variables may be primarily responsible for the effects observed.

Finally, when all the variables were entered into a regression model to examine the relative contribution of maternal and paternal depression to later child temperament, it was found that the effects of maternal depression dominated, with paternal depression seeming to have an independent effect only on the temperament of boys (see Table 6).

## 4 Discussion

Our study aimed to investigate the relationship between child temperament and parental depression in a community sample. In particular, we sought to determine the direction of any effects by using data on maternal/paternal depression and child mood and intensity collected at 2 time-points. We hypothesized that both parent to child and child to parent effects would be found. An adverse effect of paternal depression on later child temperament was seen, but appeared to be due mainly to the impact on male children. No child to father effects were evident. Clear parent to child effects were demonstrated for maternal depression, with earlier maternal depression leading to more difficult mood and intensity characteristics later on in both male and female children. This result is in line with the findings of previous research (Beck, 1996; Bruder-Costello et al., 2007; Cutrona & Troutman, 1986). There was a suggestion of child to parent effects for maternal depression, particularly in the mothers of girls, with both child mood and intensity at age 6 months predicting later maternal depression in unadjusted analyses. However this effect attenuated and was no longer significant when continuity effects were controlled for.

This study has several strengths. Data was collected from a very large sample of the population, therefore limiting selection bias. The longitudinal design allowed us to test the direction of possible effect in this large sample. The measures used, namely the EPDS and the CTS, have been well-validated, are widely used in research, and are supported by a sound theoretical basis.

There are also a number of limitations to consider. First, as is often found in studies of parents and children, the response rate was lower for fathers than mothers, indicating that there may be an element of response bias. For example, if depressed fathers were less likely to take part in the study, there may have been an underestimation of the impact of paternal depression on child temperament. Second, it was not possible to obtain clinical diagnoses of depression, and so the impact of parental depression of clinical severity on child temperament could not be ascertained. In addition, it has been suggested that the EPDS also measures anxiety (Green, 1998; Stuart, Couser, Schilder, O’Hara, & Gorman, 1998), a condition which is often co-morbid with depression, and we are therefore not able to exclude the possibility that some of our findings are in fact due to a link between parental anxiety and difficult child temperament. It should be noted, however, that the EPDS is a highly sensitive and specific tool designed primarily for the detection of depression. Third, as in many other studies, the measures of child temperament were based on maternal report. This may have led to some rater bias (higher levels of maternal depression might be related to altered perceptions of child temperament, with depressed mothers finding their children more difficult to manage). It may also have led to an overestimation of maternal effects compared to paternal effects. However, this should not have affected the comparisons of the relative direction of effect, and we were also able to control for maternal depression at the time of measurement of child temperament. Fourth, the findings with regard to direction of effect are limited to the mood and intensity dimensions of the temperament spectrum, and may not apply to other dimensions of temperament. As mentioned previously, these temperament scales were selected *a priori* as they map most closely onto the concept of positive emotionality, and previous research has shown an association between low positive emotionality in children and maternal mood disorder. It may be that different mechanisms and directions of effect apply with other areas of temperamental functioning. Finally, it is important to highlight that the first assessments of child temperament were carried out when the children were 6 months of age. It is likely that unmeasured experiences of the *in utero* and early postnatal environments will have acted prior to this time to influence children. For example, infants less than 6 months of age whose mothers are depressed have been shown to demonstrate attenuated responsiveness. One study found that 3–6-month-old infants with depressed mothers were more likely to exhibit depressive behaviour when interacting with a non-depressed person than were infants of non-depressed mothers (Field et al., 1988). EEG evidence also supports the idea that early experiences are important, with depressed mothers and their 3–6-month-old infants being more likely to have right frontal EEG asymmetry (Field, Fox, Pickens, & Nawrocki, 1995), a finding associated with a propensity to express negative emotions. Unfortunately, it was not possible within the design of our study to obtain data on very early child temperament, before some of these early, unmeasured effects could have influenced development. However, it is arguable that failing to measure these effects would only have acted to attenuate the demonstrated effect of early parental depression on later child temperament. In addition, our study did not include data on past history of parental depression. Depression tends to follow a chronic course, and it would have been interesting to compare the effects of recurrent versus first episode depressive symptoms on child outcomes. However, inclusion of this data would not be expected to have any effect on the observed directions of effect.

There are a number of potential mechanisms underlying the parent to child effects observed in this study. Genetic factors are likely to play a role and it may be that difficult temperament in infancy is an early indicator of later risk for psychiatric disorder (Bruder-Costello et al., 2007). However, the results of twin studies focusing on the effects of maternal depression and expressed emotion on child outcomes suggest that environmental factors are also important (Caspi et al., 2004; Kim-Cohen, Moffitt, Taylor, Pawlby, & Caspi, 2005). These may operate in several ways. First, parental depression may impact on the nature and quality of parent–child interactions and thereby on child temperament. It has been shown that depressed mothers demonstrate decreased sensitivity and less responsiveness towards their infants, together with more punitive parenting and less positive engagement (Murray & Cooper, 2003), and these alterations in interaction style have been associated with impaired cognitive outcomes in children (Murray et al., 1996a,b). There is more limited evidence regarding associations between paternal depression and father–child interactions (Field, Hossain, & Malphurs, 1999). Second, it has been suggested that depression could act to weaken a parent's ability to regulate the emotions of their child, potentially affecting temperamental development (Lovejoy, Graczyk, O’Hare, & Neuman, 2000). Third, parental depression may affect child temperament through more indirect effects on child environment. For example, there is a higher incidence of both marital conflict and divorce among depressed people (Briscoe & Smith, 1973; Weissman, 1987), both of which can lead to behavioural difficulties in children. The negative effects on the partner of the depressed parent may act to further increase the risks for the child.

The finding that father to child effects are specific to male children ties in with previous research (albeit on the same sample population) showing that the sons of depressed fathers are at greater risk of behavioural and emotional difficulties than the daughters of depressed fathers (Ramchandani et al., 2005). The mechanism underlying this gender difference is not clear. It could be that fathers interact differently with male children as compared to female children, or perhaps sons are more sensitive to the adverse effects of paternal depression. Further studies, focussing on observation of early father–child interaction, are necessary.

In conclusion, the results of this study suggest that child to parent effects are of marginal importance when compared to the magnitude of parent to child effects, and so the findings reinforce the importance of recognising postnatal depression in both fathers and mothers if child outcomes are to be optimised (Ramchandani et al., 2008). By means of screening and early intervention programmes, it may be possible to avoid the adverse effects of parental depression on child temperament. The nature of the optimum intervention strategy remains to be determined. Although treatments aimed at parental depression undoubtedly have benefits for the parents involved, two well-designed studies cast doubt on the idea that treatment of postnatal depression alone is sufficient to prevent adverse child outcomes. In the study of Murray et al. (Murray & Cooper, 2003; Murray, Cooper, Wilson, & Romaniuk, 2003), postnatally depressed women were assigned to routine primary care, cognitive-behavioural therapy, non-directive counselling or psychodynamic therapy at 4.5 months postpartum. Follow up at 5 years failed to show any effect of treatment on child outcome. Similarly, Forman et al. (2007) found no effect of response to treatment on child outcomes 18 months later and they suggested that therapies should also target the mother–infant relationship. One promising intervention that deserves further attention involves a home-visiting programme designed to prevent mother–infant relationship problems (van Doesum, Riksen-Walraven, Hosman, & Hoefnagels, 2008). Other methods focussing on parenting techniques and the way in which mothers perceive their infants may also prove useful in this context (Cicchetti, Rogosch, & Toth, 2000; Gelfand, Teti, Seiner, & Jameson, 1996). Finally, despite the fact that evidence for child to parent effects is limited, some parents may also benefit from schemes designed to recognise difficult temperamental characteristics in their infants; the provision of additional family support, for example in the form of nurse home visits, could prove valuable in certain cases.

## Conflict of interests

The authors have no conflicts of interest to declare.

## Figures and Tables

**Table 1:** Summary of variables used.

Variable	*N*	Minimum value	Maximum value	Mean	Standard deviation
Mood 1	10,496	0	44	15.81	5.91
Mood 2	10,325	1	48	18.08	5.69
Intensity 1	10,494	4	49	25.03	5.59
Intensity 2	10,317	4	36	21.40	4.54
Paternal depression 1	7168	0	24	3.35	3.70
Paternal depression 2	6170	0	27	3.66	3.82
Maternal depression 1	11,333	0	29	5.41	4.70
Maternal depression 2	10,401	0	30	5.72	4.80

**Table 2:** Father to child effects.

	Mean temperament score—father depressed at Time 1	Mean temperament score—father not depressed at Time 1	Beta (*p*)	Beta (*p*)^a^	Beta (*p*)^b^	Beta (*p*)^c^
Mood 2						
All	19.51	17.83	0.049 (*p* < 0.001)	0.026 (*p* = 0.084)	0.045 (*p* < 0.001)	–
Male	20.54	17.88	0.071 (*p* < 0.001)	0.054 (*p* = 0.009)	0.067 (*p* < 0.001)	–
Female	18.71	17.77	0.029 (*p* = 0.104)	0.000 (*p* = 0.995)	0.027 (*p* = 0.108)	–

Intensity 2						
All	22.29	21.24	0.038 (*p* = 0.003)	0.028 (*p* = 0.057)	–	0.027 (*p* = 0.033)
Male	23.26	21.29	0.064 (*p* < 0.001)	0.056 (*p* = 0.007)	–	0.054 (*p* = 0.002)
Female	21.53	21.18	0.013 (*p* = 0.458)	0.001 (*p* = 0.959)	–	0.003 (*p* = 0.867)

a Adjusted for paternal depression 2.

b Adjusted for mood 1.

c Adjusted for intensity 1.

**Table 3:** Child to father effects.

	Mean temperament score—father depressed at Time 2	Mean temperament score—father not depressed at Time 2	Beta (*p*)
Mood 1			
All	15.86	15.90	−0.001 (*p* = 0.923)
Male	15.66	15.88	−0.006 (*p* = 0.727)
Female	16.04	15.92	0.004 (*p* = 0.843)

Intensity 1			
All	25.12	25.01	0.004 (*p* = 0.786)
Male	25.35	25.03	0.010 (*p* = 0.590)
Female	24.91	24.99	−0.003 (*p* = 0.884)

**Table 4:** Mother to child effects.

	Mean temperament score—mother depressed at Time 1	Mean temperament score—mother not depressed at Time 1	Beta (*p*)	Beta (*p*)^a^	Beta (*p*)^b^	Beta (*p*)^c^
Mood 2						
All	20.18	17.86	0.113 (*p* < 0.001)	0.081 (*p* < 0.001)	0.081 (*p* < 0.001)	–
Male	20.70	17.91	0.133 (*p* < 0.001)	0.099 (*p* < 0.001)	0.102 (*p* < 0.001)	–
Female	19.66	17.81	0.092 (*p* < 0.001)	0.061 (*p* < 0.001)	0.058 (*p* < 0.001)	

Intensity 2						
All	22.73	21.27	0.088 (*p* < 0.001)	0.068 (*p* < 0.001)	–	0.081 (*p* < 0.001)
Male	22.83	21.34	0.088 (*p* < 0.001)	0.061 (*p* < 0.001)	–	0.074 (*p* < 0.001)
Female	22.62	21.20	0.089 (*p* < 0.001)	0.076 (*p* < 0.001)	–	0.089 (*p* < 0.001)

a Adjusted for maternal depression 2.

b Adjusted for mood 1.

c Adjusted for intensity 1.

**Table 5:** Child to mother effects.

	Mean temperament score—mother depressed at Time 2	Mean temperament score—mother not depressed at Time 2	Beta (*p*)	Beta (*p*)^a^	Beta (*p*)^b^	Beta (*p*)^c^
Mood 1						
All	16.51	15.83	0.020 (*p* = 0.001)	0.010 (*p* = 0.139)	0.000 (*p* = 0.991)	–
Male	16.29	15.74	0.015 (*p* = 0.058)	0.007 (*p* = 0.411)	−0.002 (*p* = 0.806)	–
Female	16.75	15.92	0.024 (*p* = 0.006)	0.012 (*p* = 0.194)	0.003 (*p* = 0.795)	–

Intensity 1						
All	25.48	25.03	0.014 (*p* = 0.026)	0.007 (*p* = 0.311)	–	0.005 (*p* = 0.447)
Male	25.39	25.09	0.009 (*p* = 0.292)	0.004 (*p* = 0.671)	–	0.001 (*p* = 0.916)
Female	25.58	24.97	0.020 (*p* = 0.034)	0.011 (*p* = 0.306)	–	0.010 (*p* = 0.320)

a Adjusted for maternal depression.

b Adjusted for mood 2.

c Adjusted for intensity 2.

**Table 6:** Summary of the results of linear regression models looking at the relationships between maternal and paternal depression and later child temperament.

	Maternal depression 1 (Beta (*p*))	Paternal Depression 1 (Beta (*p*))
Mood 2^a^		
All	0.053 (*p* < 0.001)	0.014 (*p* = 0.316)
Male	0.076 (*p* < 0.001)	0.043 (*p* = 0.029)
Female	0.033 (*p* = 0.115)	−0.007 (*p* = 0.723)

Intensity 2^b^		
All	0.066 (*p* < 0.001)	0.009 (*p* = 0.550)
Male	0.064 (*p* = 0.003)	0.037 (*p* = 0.076)
Female	0.071 (*p* = 0.002)	−0.017 (*p* = 0.444)

a Variables included in model: maternal depression 1; paternal depression 1; maternal depression 2; paternal depression 2; mood 1; mood 2.

b Variables included in model: maternal depression 1; paternal depression 1; maternal depression 2; paternal depression 2; intensity 1; intensity 2.
